# Quantification of Alterations in Cortical Bone Geometry Using Site Specificity Software in Mouse models of Aging and the Responses to Ovariectomy and Altered Loading

**DOI:** 10.3389/fendo.2015.00052

**Published:** 2015-04-23

**Authors:** Gabriel L. Galea, Sion Hannuna, Lee B. Meakin, Peter J. Delisser, Lance E. Lanyon, Joanna S. Price

**Affiliations:** ^1^School of Veterinary Sciences, University of Bristol, Bristol, UK; ^2^Faculty of Engineering, University of Bristol, Bristol, UK

**Keywords:** bone, mechanical loading, neurectomy, ovariectomy, aging

## Abstract

Investigations into the effect of (re)modeling stimuli on cortical bone in rodents normally rely on analysis of changes in bone mass and architecture at a narrow cross-sectional site. However, it is well established that the effects of axial loading produce site-specific changes throughout bones’ structure. Non-mechanical influences (e.g., hormones) can be additional to or oppose locally controlled adaptive responses and may have more generalized effects. Tools currently available to study site-specific cortical bone adaptation are limited. Here, we applied novel site specificity software to measure bone mass and architecture at each 1% site along the length of the mouse tibia from standard micro-computed tomography (μCT) images. Resulting measures are directly comparable to those obtained through μCT analysis (*R*^2^ > 0.96). Site Specificity analysis was used to compare a number of parameters in tibiae from young adult (19-week-old) versus aged (19-month-old) mice; ovariectomized and entire mice; limbs subjected to short periods of axial loading or disuse induced by sciatic neurectomy. Age was associated with uniformly reduced cortical thickness and site-specific decreases in cortical area most apparent in the proximal tibia. Mechanical loading site-specifically increased cortical area and thickness in the proximal tibia. Disuse uniformly decreased cortical thickness and decreased cortical area in the proximal tibia. Ovariectomy uniformly reduced cortical area without altering cortical thickness. Differences in polar moment of inertia between experimental groups were only observed in the proximal tibia. Aging and ovariectomy also altered eccentricity in the distal tibia. In summary, site specificity analysis provides a valuable tool for measuring changes in cortical bone mass and architecture along the entire length of a bone. Changes in the (re)modeling response determined at a single site may not reflect the response at different locations within the same bone.

## Introduction

Mechanical loading is the primary functional determinant of bone mass and architecture. Resident bone cells’ responses to local changes in loading-engendered strains site-specifically fine tune architectural features including curvature (1–3), eccentricity (4), cross-sectional thickness (5, 6), and polar moment of inertia, which is a measurement of bone strength (6, 7). In the young, healthy skeleton, this functional adaptation to loading matches bone’s form to its load-bearing function through a homeostatic feedback loop commonly referred to as the “mechanostat” (8, 9). This feedback loop maintains bone strains within a tolerated range during habitual levels of loading. However, the mechanostat appears to fail in humans with age, correlating with reduced availability of estrogen leading to the bone fragility characteristic of osteoporosis (10, 11). Thinning of the load-bearing cortices predisposes to fractures, which in long bones occur preferentially at specific sites including the hip and wrist. It is now well documented that the overall geometry of these high-risk sites, particularly of the femoral neck, determines bone resistance to fracture independently of the absolute mass of bone present (12–14).

Despite regional differences in mass and architecture along their length, long bones must locally fine tune their structure to withstand load-bearing as functional units. At the cellular level, this is achieved through modeling (in which the activity of bone forming osteoblasts is independent of resorbing osteoclasts) and re-modeling (in which osteoblast activity follows osteoclastic resorption). Bone mass and architecture in one region influence the mechanical strain environment in other regions (15), such that structural properties at different sites within the same bone are inter-related. For example, the angle of articulation between the femur and tibia predicts the location of bisphosphonate-associated atypical fractures in the femoral diaphysis in humans (16). Experimental studies in rodents using micro-computed tomography (μCT) and histomorphometry analyses have documented the bone formation response following physiological axial loading and demonstrated that it is site specific. For example, in the ulna loading model, an adaptive response is observed distally but not proximally (17) and axial loading in the mouse tibia elicits a response at 37% of the bone’s length from the proximal end, but not distally at 75% of the bone’s length (6, 18, 19). Consequently, deductions on the effect of loading based on measurements at a single cortical site cannot be generalized to the rest of the bone. This is particularly relevant given the increasing use of *in vivo* μCT scanning of short sections of bone to provide longitudinal data within the same bone.

Unlike the site-specific targeting of bone (re)modeling following loading, systemic and genetic interventions may have indiscriminate, uniform effects on bone mass and architecture and where load-bearing remains constant may intrinsically alter the strain environment. The long-term (re)modeling outcomes of osteogenic or catabolic interventions will therefore be influenced by their interactions with the mechanostat, either suppressing bone’s adaptation to loading such that net bone loss occurs or enhancing the sensitivity/responsiveness of the mechanisms involved in the mechanostat such that a lower level of habitual strain is tolerated (18, 20). Since systemic interventions are additive, synergistic, or oppose the site-specific effects of loading, their outcome is expected to have a degree of regionality. This is clearly demonstrated in our recent report that deletion of *Prkc*α in mice leads to age-, gender-, and site-specific alterations in bone structure under the influence of mechanical load-bearing (21). Documentation of these findings in *Prkc*α*^−/−^* mice required systematic analyses of bone mass and architecture at numerous sites. However, there are limited tools available for analyzing changes in whole-bone cortical architecture following altered loading or systemic interventions such as ovariectomy.

The primary aim of this study was to generate, validate, apply, and freely disseminate a new site specificity software tool, which allows bias-free and convenient quantification of standard measures of bone mass and architecture at multiple cortical bone sites in the mouse tibia. To demonstrate the efficacy of this software, we produced global maps of changes in bone geometry along the length of the mouse tibia in a number of routinely investigated experimental interventions in bone biology research (aging, axial loading, ovariectomy, and disuse).

## Materials and Methods

### Site specificity software for the analysis of μCT data

Mouse tibiae were scanned with high resolution μCT (Bruker, Kontich, Belgium) with a voxel size of 4.8 μm as previously reported (6, 19). Images were reconstructed in NRecon (Bruker, Kontich, Belgium) with the following settings: threshold 1.000–1.160, ring artifacts correction 5, beam hardening correction 25%, smoothing 0. Folders containing all the sequentially labeled cross-sectional images from a single bone are defined as the “in path” for site specificity analysis. A new, empty folder labeled “output” is created inside each “in path” folder. The newly developed site specificity program (Data Sheet 1 in Supplementary Material) is then opened for editing in MatLab (R2012a). The site specificity software “in path” (line 13) is set to the folder containing the cross-sectional μCT images of the bone to be analyzed following the format in the example provided (Table S1 in Supplementary Material, Excel file containing a worked out example and a blank sheet into which raw values generated below can be entered). This program is then run as a standard script in MatLab to identify and analyze the μCT slices corresponding to each 1% of the bone’s length.

**Figure 1:**
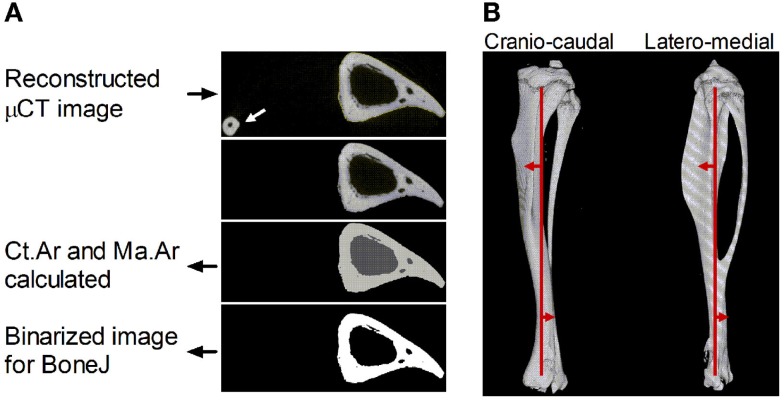
**Description of the sequential steps in the site specificity method**. **(A)** Reconstructed μCT images in the “in path” folder are automatically selected to analyze images corresponding to each 1% site along the bone’s length based on the total number of image files in the folder. The fibula (arrow) is excluded by selecting the largest continuous shape. The software then calculates cortical area (Ct.Ar, light gray) and medullary area (Ma.Ar, dark gray) and collates these values for each site in a single .csv file. Binarized images corresponding to each 1% site are also saved for BoneJ analysis. **(B)** Reconstructed 3D image of a μCT-scanned bone section through the cranio-caudal (i.e., anterior–posterior *X*) axis and latero-medial (*Y*) axis with the central line approximately indicated running through the cross-sectional center of gravity proximally and distally (vertical red line). The direction of centroid deviation is indicated by the red arrows showing curvature anterior to the central line proximally and posterior curvature distally, crossing the midline at approximately 70% of the bone’s length from the proximal end.

Measures of area given are in pixels, with each pixel representing the scanning voxel size (in our system each pixel represents 4.8 μm). Site specificity isolates the tibia and calculates the area of bone (cortical area, Ct.Ar) and the enclosed non-bone area (marrow area, Ma.Ar, Figure 1A) by segmenting the pixel values using the *k*-means algorithm applied to pixel intensity values where *k* is equal to 2. For pixels representing a mixture of tissue types (partial volume effect), their initial classification depends solely on their similarity to the mean of the two clusters. However, in a subsequent processing stage, pixels disconnected from the main bony component are classified as background using connected component analysis. Summation of Ct.Ar and Ma.Ar provides tissue area within the periosteum (Tt.Ar). In cases in which the cortex is breached such that there is no enclosed space, neither Ma.Ar nor Tt.Ar can be calculated for that individual slice but Ct.Ar is still reliable. This is relatively rare in normal mouse tibiae given vessels and cortical defects rarely run directly through the entire cortex in a single cross-section. Errors increase toward the proximal (trabecular bone) and distal (calcaneus) extremes of the bone such that only the 81 individual slices 4.8 μm thick at each 1% site between 10 and 90% of the bones length were analyzed.

The site specificity program collates all the measures in an Excel (.csv) file within the “output” folder. In addition, it saves binarized images of the tibia without the fibula in the “output” folder as .gif files, which retain the same pixel size (Figure 1A) but have a much smaller file size, facilitating subsequent analysis and storage. These .gif files can be exported to the previously validated and freely available BoneJ program in ImageJ (22, 23) to obtain cross-sectional thickness (Cs.Th), moments of inertia (minimum moment of inertia IMin, maximum moment of inertia Imax, and polar moment of inertia PMI through the summation of IMin and IMax), cross-sectional centroid in the *X* and *Y* direction. BoneJ also calculates Feret’s maximum diameter, minimum diameter, and angle by rotating the cross-sectional images to identify the maximum and minimum caliper dimensions. Eccentricity was obtained from the maximum and minimum diameters essentially as calculated by Bruker software (24) based on the length of the major axis (Feret Max from BoneJ) and minor axis (Feret Min from BoneJ).

All bones were oriented such that Feret’s angle at the 37% site (arbitrarily chosen) was 0°, i.e., parallel to the *x*-axis such that the *x*-axis is anterior–posterior while the *y*-axis is medial–lateral. Centroid was then used to calculate absolute center of mass deviation from the bone’s central axis as a measure of curvature. The central axis was defined as an extrapolated straight line running between 5 and 95% of the bone’s length (Figure 1B). Calculating the absolute deviation from this line defined within the bone itself accounts for any differences in bone position while being scanned, producing values, which were highly consistent between similar mice in a semi-automated manner but which lose information on the direction of curvature along the axis being investigated.

The outputs of the site specificity program are: Ct.Ar, Ma.Ar, Tt.Ar, and binarized images, which can then be analyzed with BoneJ in ImageJ to provide Cs.Th, PMI, eccentricity, and curvature in the anterior–posterior (*x*) or medial–lateral (*y*) directions. Only cortical bone can be analyzed using site specificity. The site specificity and BoneJ analyses for an entire tibia with the data entry form provided (Table S1 in Supplementary Material) take <10 min on a standard laptop computer.

### Site specificity validation

In order to ensure that bone structural measurements obtained through site specificity analysis are directly comparable with “conventional” μCT analyses, cortical parameters were calculated at four sites in each of six mice using conventional μCT or site specificity analysis. Conventional μCT analysis was performed using CTan (Bruker, Kontich, Belgium) as previously reported by our group (6, 19). In brief, bones were imaged with an X-ray tube voltage of 49 kV and current 200 μA, with a 0.5 mm aluminum filter. The scanning angular rotation was 180° and the angular increment was 0.6°. The voxel size was 4.8 μm isotropically. Images were reconstructed using a modified Feldkamp algorithm in NRecon and opened for analysis in CTan. The regions of interest calculated based on the length of each bone. A region of 100 μCT slices around each region of interest was selected and the fibula manually excluded (versus automatic exclusion using site specificity). Reconstructed cross-sectional gray scale images were segmented into binary images using adaptive local thresholding with a threshold window of 100–255. The images then underwent sequential modifications to reduce noise and improve continuity and then image series underwent removal of white speckles (<100 pixels area) and black speckles (<20 voxels). The outer periosteal border was isolated by a shrink wrap function stretching over holes with a diameter greater than 10 pixels. The resulting standard cortical bone measures averaged over 100 slices were compared with the Ct.Ar, Tt.Ar, and Ma.Ar values automatically generated by the site specificity software at the single cross-sectional slice at the desired percentage site.

To test the reproducibility of site specificity analysis, a bone was analyzed five times with complete system restarts between analyses. In addition, Ct.Ar calculated by site specificity was compared to the same parameter calculated by BoneJ.

### Animal studies

To investigate the effect of aging, young adult 18-week-old and aged 19-month-old C57B∖ 16 mice were obtained from Charles River (*n* = 6 in each age group).

To study the effect of loading and disuse, C57B/16 mice were also obtained from Charles River. For loading experiments, 17-week-old female mice (*n* = 15) were subjected to axial tibial loading of the right limb three times a week for 2 weeks as previously described (6, 19). In brief, the flexed knee and ankle joints are positioned in concave cups; the upper cup containing the knee is attached to an actuator arm of a loading device and the lower cup to a dynamic load cell. The tibia is held in place by a 0.5 N continuous static pre-load. Forty cycles of dynamic load are superimposed with 10 s rest intervals between each cycle. The protocol for one cycle consists of loading to the target peak load, hold for 0.05 s at the peak load, and unloading back to the 0.5 N pre-load at a load of 13.5 N to engender a peak strain of 2,250 με on the medial surface of the tibia 37% of its length from the proximal end. The left limbs served as normally loaded internal controls. Disuse was induced as previously described (6, 25) through unilateral sciatic neurectomy (SN) (*n* = 6) of the right limb of 17-week-old female mice. Bones were collected 3 weeks following SN. The left limbs served as normally loaded internal controls.

To investigate the effect of estrogen withdrawal, ovariectomy (OVX) was performed as previously described (18, 21) in 8-week-old mice C57Bl/6 mice bred in house. Bones were collected 10 weeks after ovariectomy and compared to non-ovariectomized littermate controls (*n* = 6).

All procedures complied with the UK Animals (Scientific Procedures) Act 1986 undertaken under project license PPL 30-2829 and were reviewed and approved by the University of Bristol ethics committee (Bristol, UK). All mice were allowed free access to water and a maintenance diet containing 0.75% calcium (EURodent Diet 22%; PMI Nutrition International, LLC, Brentwood, MO, USA) in a 12-h light/dark cycle, with room temperature at 21 ± 2°C. Mice were housed in groups of up to five animals and all cages contained wood shavings, bedding, and a cardboard tube for environmental enrichment.

### Statistics

Statistical comparison was by mixed model analysis in SPSS (PASW Statistics, v.18) with bone site as a fixed categorical parameter, the intervention (aging, loading, disuse, OVX, or control) as a fixed effect, and an intervention by site interaction to determine whether the effect of the intervention was site-specific (i.e., significantly different at different sites) or uniform. For loading and disuse analyses, mouse ID was included as a random effect to account for left and right limbs originating from the same mouse. When the effect of the intervention was significant overall, a *post hoc* Bonferroni correction was applied to identify the individual sites at which the effect was significant at *p* < 0.05.

In order to test the ability to detect differences at *p* < 0.05, a simulated 10% global changes in Ct.Ar were statistically analyzed using mixed models. This magnitude of change was selected because OVX caused a −10.0 ± 0.03% change in Ct.Ar averaged across all sites (range −4 to −20%), described in the Section “Results.” To do this, Ct.Ar values from six mice were increased by 10% across all sites (the “Intervention”) and analyzed by mixed model analysis with *post hoc* Bonferroni. The overall *p* value associated with the intervention fixed effect and the *p* values comparing each site were then obtained.

Results are presented as the mean ± SEM.

## Results

### Validation of the site specificity program

**Figure 2:**
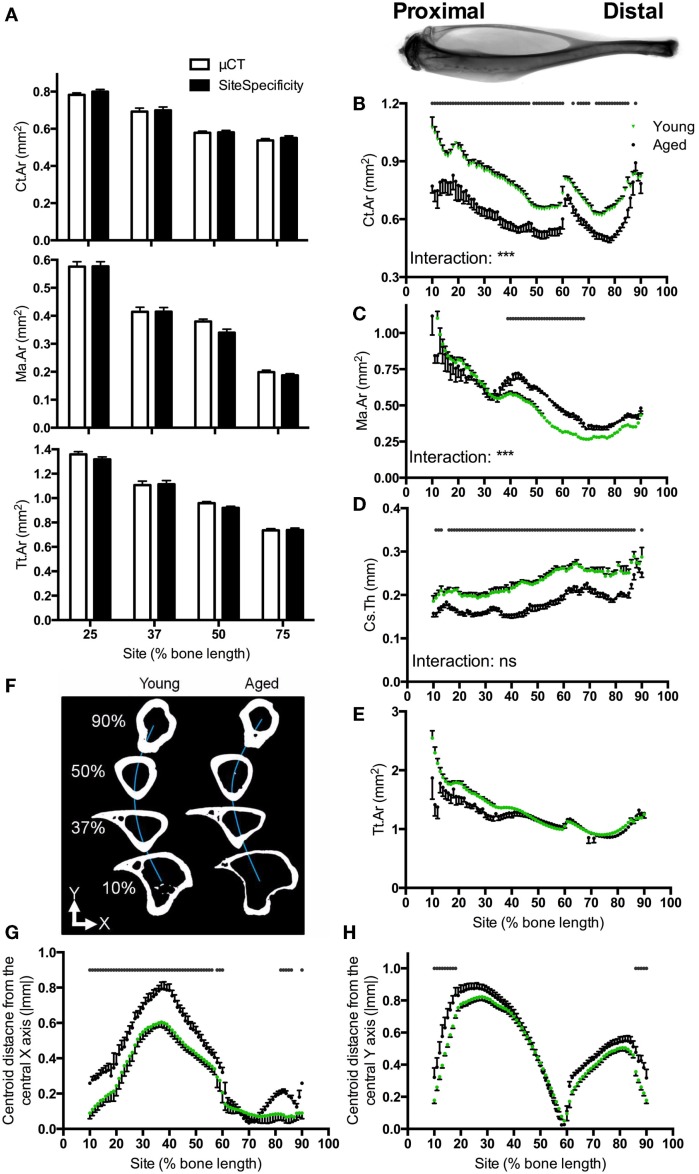
**Software validation and application to compare bones from young and aged mice**. **(A)** Comparison of Ct.Ar, Ma.Ar, and Tt.Ar values obtained from six mice at the four indicated sites using site specificity analysis or conventional μCT analysis illustrating direct comparability between these two methods. Inset: radiograph of a tibia and fibula from a young mouse approximately aligned to the corresponding site values in the graphs below. **(B)** Ct.Ar, **(C)** Ma.Ar, **(D)** Cs.Th, and **(E)** Tt.Ar were calculated for bones of young and aged mice. **(F)** Schematic representation looking down the tibia of a young and aged mouse approximately illustrating the deviation in medial–lateral curvature (blue line, *Y*). **(G)** Absolute anterior–posterior and **(H)** medial–lateral centroid deviation in tibiae from young and aged mice. Points represent the mean ± SEM, *n* = 15 young, and 6 aged. Dots/bars above the graphs indicate sites of significant difference, *p* < 0.05 following Bonferroni correction. Interactions are the site by aging interactions calculated by mixed models; ns not significant, ***p < 0.001.

Ct.Ar, Ma.Ar, and Tt.Ar measures obtained from site specificity and conventional μCT analysis were highly correlated (*R*^2^ = 0.99 for each parameter, Figure 2A), with slight differences between the two likely due to conventional μCT analysis taking the average of 100 CT slices, whereas site specificity analysis analyses individual slices corresponding to each 1% site. Within each site tested, the site specificity value obtained from a single slice will always be different from that generated by conventional μCT analysis if the parameter in question changes rapidly over the 100 lines analyzed. In areas of rapid change, such as at the tibia–fibula junction, the site specificity represents the actual value for the cross-sectional image corresponding to that level rather than an average of slices around the level of interest. Using the statistical approach described, a simulated intervention effect causing 10% change in Ct.Ar could be detected as significant (*p* < 0.05) with *n* = 4 bones. With a sample size of *n* = 6 (the smallest group size used in the studies presented here), a 10% change was detected as significant in 98.8% of sites analyzed.

Parameters obtained from BoneJ were as previously validated (22) and the eccentricity parameter calculated from these was also highly correlated to values obtained from conventional μCT analysis (*R*^2^ = 0.96, not shown). Results obtained using this software can therefore be directly compared to data obtained through conventional μCT analysis. Re-analysis of the same bone produced identical results each time (*R*^2^ = 1, *t*-test *p* = 1, not shown). Ct.Ar calculated by site specificity and BoneJ were identical (*R*^2^ = 1, *t*-test *p* = 1, not shown).

Site specificity analyses illustrate differences in structural parameters along the length of the bone functional unit. The proximal tibia of young mice has a greater cortical area surrounding a larger medullary area, but a narrower cortical thickness as compared with distal regions of the same bone, such that total tissue area is smaller distally than proximally (Figures 2B–E). Absolute centroid deviation from the central axis, as a measure of bone curvature, in the anterior–posterior direction is more marked in the proximal to middle region of the bone corresponding to the tibial crest (Figures 2F,G). Curvature in the medial–lateral direction is also most marked in the proximal half of the bone, curving back to the midline by 60% of the bone’s length from the proximal end (Figure 2H). All patterns of curvature and structural parameters were highly consistent between different mice: for example, the tibia–fibula junction occurred within 2% of the bone’s length in all young mice analyzed.

### Effect of aging

Aging was associated with smaller cortical area and larger medullary area, but these differences were site-specific such that the greatest differences were observed in the proximal regions of the bone (Figures 2B,C). Conversely, bones from aged mice had lower cortical thickness than those from young mice, but this difference was uniform along the length of the bone (Figure 2D). Differences in total tissue area between young and aged were not significant (Figure 2E).

Aging was associated with significant differences in deviation in centroid from the central axis. Centroid deviation was greater in aged than young adult bones in the anterior–posterior axis, particularly in the proximal half of the bone (Figure 2G). Centroid deviation along the medial–lateral axis was greater in aged bones in both the proximal and distal extremes of the region analyzed (Figure 2H).

### Effect of additional loading

**Figure 3:**
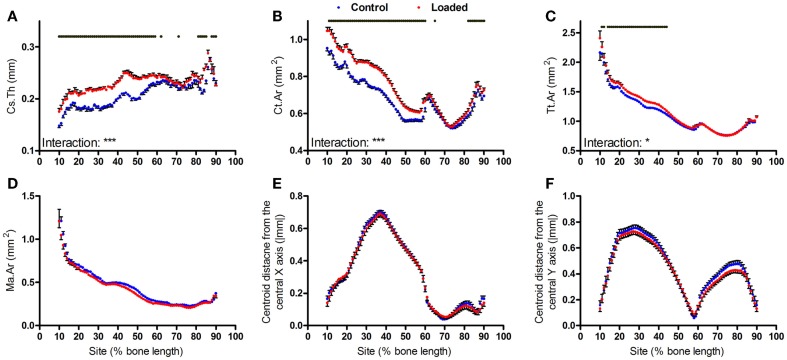
**Analysis of response of the mouse tibia to loading at each site along the bone’s length**. Site specificity analysis of **(A)** Cs.Th, **(B)** Ct.Ar, **(C)** Tt.Ar, **(D)** Ma.Ar, **(E)** absolute anterior–posterior, and **(F)** medial–lateral centroid deviation calculated from control left tibiae and loaded right tibiae from the same mice, *n* = 15. Dots/bars above the graphs indicate sites of significant difference, *p* < 0.05 following Bonferroni correction. Interactions are the site by aging interactions calculated by mixed models; *p < 0.05, ***p < 0.001.

We have previously reported that the region of the mouse tibia, which undergoes the greatest osteogenic response following non-invasive axial loading, is localized to 37% of the bone’s length from the proximal end, whereas other studies using similar models have analyzed responses at the mid-diaphysis (26–30). Loading increased cortical thickness and cortical area at both sites, whereas total tissue area was only significantly increased more proximally (Figures 3A–C). Although the effect of loading on medullary area was significant overall (mixed model *p* = 0.01), and left versus right comparisons were significant (*p* < 0.05) by *t*-test at all sites between 40 and 70% of the bone’s length, none of these differences were statistically significant following Bonferroni correction for multiple comparisons (Figure 3D). Thus, a study analyzing the effect of loading on medullary area would conclude this as reduced if the 50% site is analyzed, but that it is unchanged if the 37% site is analyzed (Figure S1 in Supplementary Material).

Two weeks of loading did not significantly alter bone centroid deviation in either the anterior–posterior or medial–lateral directions (Figures 3E,F).

### Effect of disuse

**Figure 4:**
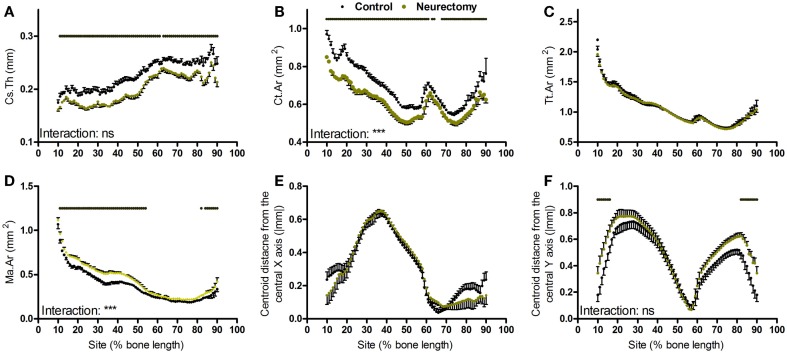
**Analysis of response of the mouse tibia to neurectomy-induced disuse at each site along the bone’s length**. Site specificity analysis of **(A)** Cs.Th, **(B)** Ct.Ar, **(C)** Tt.Ar, **(D)** Ma.Ar, **(E)** absolute anterior–posterior, and **(F)** medial–lateral centroid deviation calculated from control left tibiae and disused right tibiae from the same mice, *n* = 6. Dots/bars above the graphs indicate sites of significant difference, *p* < 0.05 following Bonferroni correction. Interactions are the site by neurectomy interactions calculated by mixed models; ns not significant, ***p < 0.001.

Bones’ response to changes in loading follows a linear continuum between the low strains association with the bone loss and the higher strains associated with bone gain (6). However, unlike loading, SN-induced disuse resulted in uniform loss of cortical thickness throughout the tibial cortex (Figure 4A). The reduction in bone area due to disuse was site specific; greater in the more proximal than distal regions of the bone (Figure 4B). Disuse did not alter total tissue area but significantly increased marrow area in a site-specific manner (Figures 4C,D). Curvature in the anterior–posterior axis was not significantly altered by disuse whereas medial–lateral curvature was significantly increased at the proximal and distal extremes of the region analyzed, but the site by neurectomy interaction was not statistically significant (Figures 4E,F).

### Effect of ovariectomy

**Figure 5:**
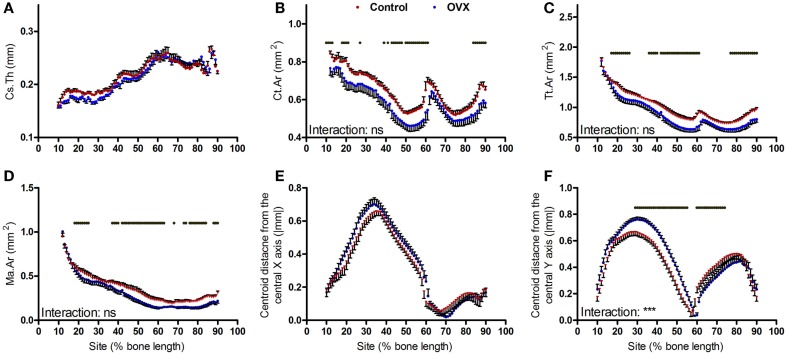
**Analysis of effect ovariectomy on the mouse tibia at each site along the bone’s length**. Site specificity analysis of **(A)** Cs.Th, **(B)** Ct.Ar, **(C)** Tt.Ar, **(D)** Ma.Ar, **(E)** absolute anterior–posterior, and **(F)** medial–lateral centroid deviation calculated from left tibiae of non-ovariectomized control mice and left tibiae from ovariectomized mice, *n* = 6. Dots/bars above the graphs indicate sites of significant difference, *p* < 0.05 following Bonferroni correction. Interactions are the site by ovariectomy interactions calculated by mixed models; ns not significant, ***p < 0.001.

Ovariectomy during growth did not alter average cortical thickness achieved in adulthood (Figure 5A) but resulted in smaller cortical bone area in a uniform manner (Figure 5B). This involved smaller total tissue area and larger medullar cavity in a non-site-specific manner (Figures 5C,D). Ovariectomy did not alter the absolute centroid deviation in the anterior–posterior axis (Figure 5E), but significantly increased medial–lateral centroid deviation between approximately 30 and 60% of the bone’s length while decreasing it between approximately 65 and 80% of the bone’s length from the proximal end (Figure 5F). This pattern of change in bone curvature is distinct from that observed following aging or disuse.

### Site-specific influences on polar moment of inertia and eccentricity

**Figure 6:**
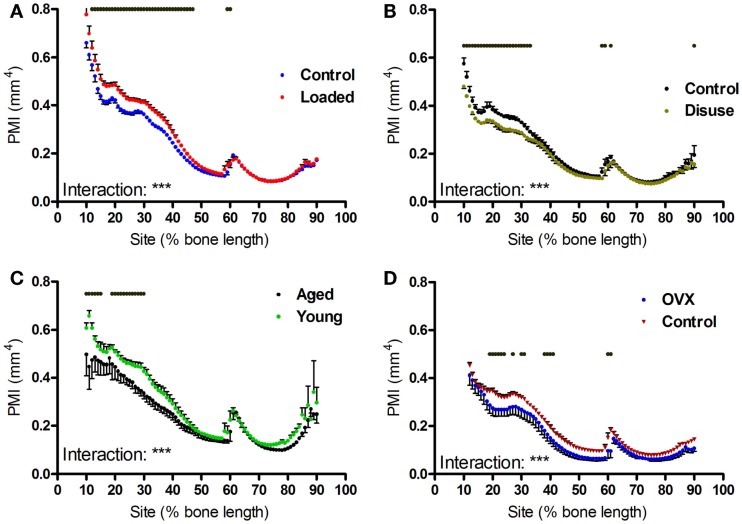
**Effects of loading, disuse, aging and ovariectomy on polar moment of inertia at each site along the mouse tibia**. PMI was calculated at each 1% site along the length of **(A)** control left and loaded right tibiae, **(B)** control left and neurectomized right tibiae, **(C)** left tibiae of aged and young mice, **(D)** left tibiae of control and ovariectomized mice. Dots/bars above the graphs indicate sites of significant difference, *p* < 0.05 following Bonferroni correction. Interactions are the site by intervention interactions calculated by mixed models; **p < 0.01, ***p < 0.001.

Aging, axial loading, and ovariectomy all influence overall strength of the bone functional unit. Moments of inertia are structural parameters related to bone strength, which can be calculated from CT images (31). Within each tibia, the polar moment of inertia was greater in the proximal region of the bone than the distal regions beyond the tibia–fibula junction (Figures 6A–C). Loading increased polar moment of inertia in a site-specific manner, with significant changes proximally but not distally (Figures 6A–F). Tibiae from aged mice followed a similar pattern, with smaller polar moment of inertia in the proximal but not distal regions of the bone relative to young mice (Figures 6G–I).

Unexpectedly, ovariectomy also decreased polar moment of inertia in a site-specific manner (Figures 6J–L), despite having caused a uniform loss in bone area. We hypothesized that the site-specific effects on polar moment may be due to uniform bone loss from different baselines along the length of the bone functional unit. Simulated reductions in bone mass at the periosteal or endosteal surface cause relatively greater reductions in polar moment of inertia at the proximal 37% site than at the distal 75% site (Figure S2 in Supplementary Material), indicating that the polar moment of inertia in the distal tibia is relatively unaffected by changes in cortical area compared with the less circular proximal tibia. This suggests that the uniform reduction in bone area caused by ovariectomy would reduce PMI in the proximal tibia to a greater extent than in the distal tibia.

**Figure 7:**
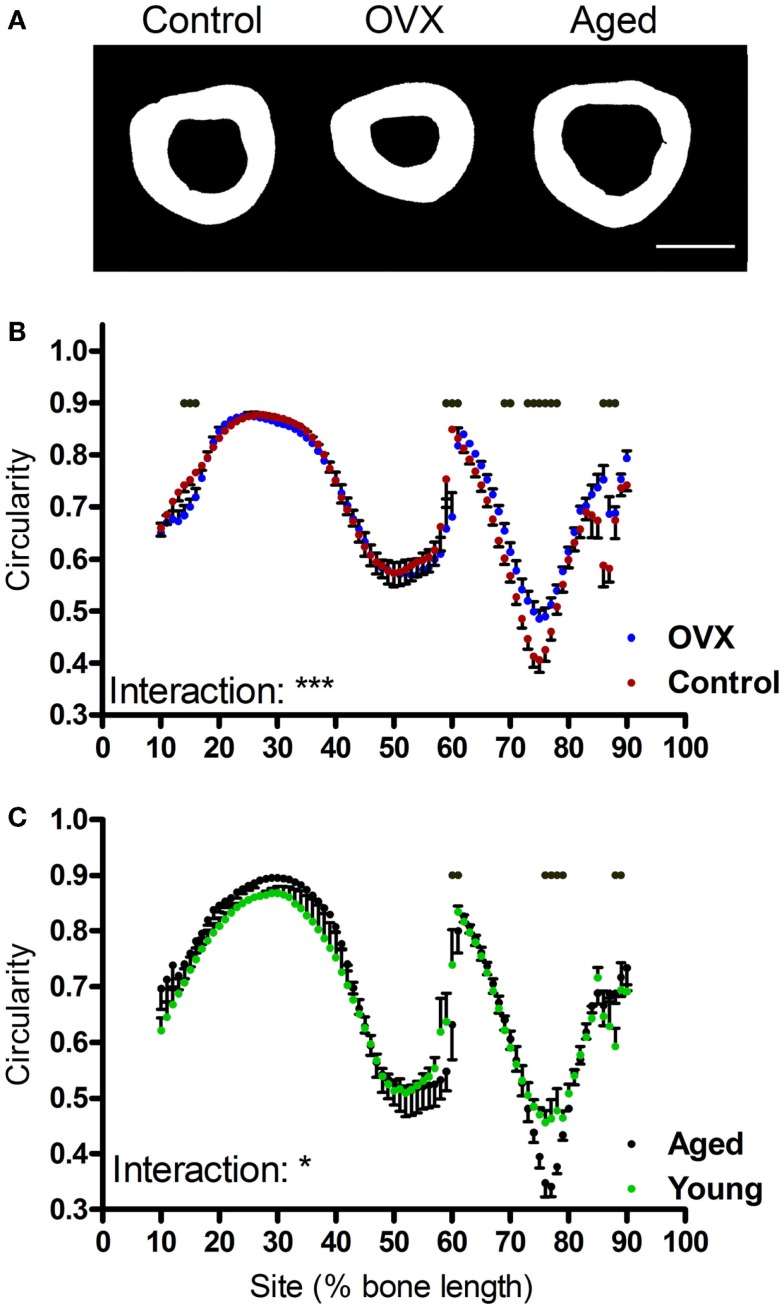
**Effects of ovariectomy and aging on the eccentricity of the mouse tibia at each site along the bone’s length**. **(A)** Representative binarized images of the cross-sections through the 75% site of the tibia from a young control mouse, an ovariectomized mouse, and an aged mouse. **(B,C)** Eccentricity calculated from left tibiae of **(B)** non-ovariectomized control mice and left tibiae from ovariectomized mice or **(C)** young or aged mice, *n* = 6. Dots/bars above the graphs indicate sites of significant difference, *p* < 0.05 following Bonferroni correction. Interactions are the site by ovariectomy interactions calculated by mixed models; *p < 0.05, ***p < 0.001.

Given moments of inertia influenced by shape, we also investigated whether the interventions tested altered bone shape by calculating the eccentricity parameter, which is a load-bearing responsive geometric parameter (4). Eccentricity was not significantly altered by loading or disuse (not shown), but was significantly affected by both ovariectomy and aging in a site-specific manner predominantly in the distal regions of the bone (Figures 7A–C).

## Discussion

Bone mass and architecture are the outcome of bone’s functional adaptation to local load-bearing occurring on a background of systemic and genetic influences. The effect of these influences can be additive, synergistic, or to oppose those of functional adaptation. The study that we report here describes alterations in bone mass and architecture resulting from aging, changing the mechanical environment, or ovariectomy. It shows that analysis of cortical bone at multiple sites provides more meaningful information than that obtained from analysis of candidate sites because several parameters are altered in a site-specific manner and so conclusions made about changes in one site may not be meaningfully extrapolated to the remainder of the bone functional unit. This is particularly true in relation to the moments of inertia and curvature, which can only be appreciated when the entire bone is analyzed. These analyses have shown that site specificity analysis provides additional information over conventional approaches and have demonstrated that several physiological contexts, not only mechanical loading, can induce significant site-specific changes in cortical bone mass and architecture. Site specificity analysis quantifies standard cortical parameters precisely and reproducibly. It provides measures, which are directly comparable to those obtained using conventional μCT analysis. Furthermore, using mixed model statistical approaches, it allows comparisons at numerous sites along the bone’s length with adequate statistical power.

Most published studies investigating the effects of altered mechanical loading, systemic or genetic interventions on bone only investigate differences at pre-determined sites along the bone’s length. This may begin to explain discrepancies in reported responses. For example, the present study using a relatively large sample of 15 mice suggests that axial loading of the mouse tibia tends to reduce marrow area if the 50% site is analyzed, but does not significantly alter marrow area at the 37% site in the same mice. This is consistent with previous studies analyzing the 50% site concluding that axial loading alters the endosteal surface (26–28), whereas studies focused on the 37% site observed no such change (29, 30, 32). It is now widely accepted that changes in local mechanical loading have site-specific effects and it is common practice to compare responsive and less responsive sites along the bone’s length (6, 20, 25, 33). The novel methodology described here eliminates the intrinsic bias in selecting candidate sites irrespective of potential inter-group differences in properties such as curvature, and facilitates comparisons by allowing automated determination of cross-sectional parameters at each 1% of the bone’s length. Curvature and eccentricity are likely to be influenced by poorly understood interactions between the mechanical, genetic, and hormonal contexts, which ultimately determine bone architecture. For example, in humans, the buckling ratio of the femoral neck is a macro-architectural feature, which deteriorates with age (34, 35) and is influenced by genetic polymorphisms including variants in the paracrine signaling molecule Wnt16 (36–38). Understanding how the magnitude and direction of mechanical cues informs overall bone structure is likely to require integration of biomechanical and mechanistic studies. However, characterization of site-specific and macro-architectural bone phenotypes in transgenic mouse models may begin to elucidate the genetic determinants of bone architecture beyond simply quantifying bone mass at individual sites.

To this end, a few groups have developed systems to map differences in bone mass between wild-type and transgenic mice to specific locations (33, 39, 40). In addition, commercially available software is also able to analyze serial 2D μCT images, as we have previously reported (21). A key advantage of site specificity over currently available software is the ability to automatically analyze images based on percentages of bone length (represented by the absolute number of cross-sectional images for that bone) rather than analyzing specific lengths. The percentage-based approach accounts for differences in bone length and thereby facilitates alignment between different mice. Another advantage of site specificity is the free availability of the program code provided with this publication, such that it can be adapted to novel approaches. In addition, automated site specificity analysis is very rapid: in our hands, an entire bone can be analyzed on a laptop computer in 4 min (BoneJ analyses being additional) as compared with analysis of a single 100-line section using commercially available software taking 5–6 min. Irrespective of the software used, the general method of analyzing μCT data at multiple sites may begin to inform the mechanisms by which bones achieve and maintain their geometry.

Biological regulation of macro-architectural features is suggested by consistency in properties such as the position of the tibia–fibula junction between different mice. The genetically determined baseline toward which the diaphyses of long bones are programed to develop in the absence of mechanical stimulation is effectively cylindrical (41, 42). Although cylindrical bones would be ideally suited to withstand loading applied in a predictably axial direction, this design is inefficient when the direction of loading cannot be reliably predicted as in the case of footfall. Consequently, bone (re)models toward a compromise between energetically unfavorable increases in mass and functionally integral resistance to fracture. This compromise has been suggested to explain the tendency of bones to be curved; although curvature is inefficient in that it increases strains experienced, it may be beneficial by providing predictability of strain direction (15, 43).

The biomechanical significance of changes in curvature observed following disuse, ovariectomy, and aging, but not following loading in the accustomed axial direction, is poorly understood. As curvature of the mouse tibiae provides a damping effect (15), increased curvature may be an adaptive process in situations of sub-optimal bone mass. This hypothesis is consistent with the observation that the proximal region of the mouse tibia, which has the greatest deviation from the central axis, also has greater mass and is less cylindrical than the distal tibia. The greater polar moment of inertia in the proximal than distal tibia suggests greater resistance to torsional forces (31). Similar regional differences in polar moment of inertia have been reported in the human tibia (44). Finite element modeling may be able to further clarify whether proximal tibial architecture increases distal strain predictability and thereby favors a more cylindrical shape. Given simulated periosteal or endocortical expansion has a greater absolute effect on the calculated polar moment of inertia in the proximal than distal tibia, it is possible that the apparent “unresponsiveness” of polar moments of inertia in the distal tibia following any of the interventions tested may be due to these changes being too small to detect as statistically significant. When effect sizes are small, performing multiple comparisons followed by a Bonferroni *post hoc* test results in Type II errors (at a rate of 1.2% in our simulated 10% change in Ct.Ar with *n* = 6). Future studies aimed at detecting smaller differences may require an increase in sample size to maintain their statistical power. Alternatively less stringent *post hoc* tests (e.g., Tukey’s HSD) could be used while accepting a greater proportion of false positive results. Another approach would be to analyze a smaller subset of the data; for example, by only statistically comparing each 5% site to reduce multiple comparisons. Whatever approach is used, graphical representation of site specificity outputs provides a visual representation of the magnitude of differences in different sites, permitting more targeted investigation of sites of potential interest; e.g., by histomorphometry.

Notwithstanding, the distal tibia below the tibia–fibula junction is not “architecturally inert.” In a recent study, electrical stimulation of the rat common peroneal nerve resulting in muscle contraction was found to cause bone formation in this distal region of the tibia, correlating with the highest localized strain levels predicted by finite element modeling (45). In the present study, significant differences were observed in distal tibial eccentricity following ovariectomy and aging. Although 2 weeks of disuse has previously been reported to decrease bone eccentricity in growing mice (4), 3 weeks of disuse did not result in significant changes in eccentricity in skeletally mature mice in the present study. Also in growing mice, 10 weeks of ovariectomy significantly increased eccentricity in the distal tibia, potentially reflecting altered loading due to impoverished architecture proximal to the tibia–fibula junction. Aging had the opposite effect, decreasing eccentricity as the distal tibial cortex thinned into a more uniformly circular shape with age.

Aging is associated with reduced bone strength, thinning of the load-bearing cortices, and impoverished bone architecture in mice (19, 26) as in humans (12, 13, 34, 46). Why it is that functionally adapted bones undergo seemingly maladaptive age-related structural changes remains incompletely understood. At the cellular level, age-related deficiencies in the responses of osteoblasts to mechanical strain have been identified (19). At the tissue level, we describe in this study the site-specific reduction in cortical area and increase in medullary area as well as uniform reduction in cortical thickness in the mouse tibia, a pattern of change, which is similar to that seen following neurectomy. These similarities between the effects of aging and neurectomy-induced disuse are consistent with our group’s hypothesis that aging reflects a failure of bone’s adaptation to mechanical loading such that (re)modeling occurs as in a state of disuse (10).

In summary, automated site specificity analysis of bone structure at multiple sites along a bone’s length provides the opportunity to study local and systemic influences on site-specific responses, which alter bone architecture. Application of this system of analysis to the mouse tibia demonstrates that increased or decreased loading, ovariectomy, and aging all cause site-specific changes in bone, such that the structural alterations achieved by these changes, be they strategic or maladaptive, remain preferentially targeted to specific locations. Even when the imposed (re)modeling stimulus is apparently systemic, as in the case of ovariectomy, alteration in bone structural properties including polar moment of inertia remains site specific. Elucidating the interactions between systemic and mechano-adaptive (re)modeling stimuli, which determine bone architecture, and therefore fracture resistance is expected to be facilitated by integrated analysis of structural responses along the length of the bone functional unit. Site specificity analysis may help refine experimental design by selectively targeting optimally responsive or unresponsive cortical regions. It is anticipated that application of site specificity analysis will begin to identify mechanisms by which genetic or systemic cues enhance or suppresses the local, site-specific mechanisms underlying the mechanostat to determine the overall mechanical suitability of the bone functional unit.

## Conflict of Interest Statement

The authors declare that the research was conducted in the absence of any commercial or financial relationships that could be construed as a potential conflict of interest.

## Supplementary Material

The Supplementary Material for this article can be found online at http://www.frontiersin.org/article/10.3389/fendo.2015.00052/abstract
