# Supplementary material for: Quantification of Alterations in Cortical Bone Geometry Using Site Specificity Software in Mouse models of Aging and the Responses to Ovariectomy and Altered Loading
Source: Front Endocrinol (Lausanne). 2015 Apr 23;6:52. doi: 10.3389/fendo.2015.00052 (PMC4407614; doi:10.3389/fendo.2015.00052)
Supplement: Supplementary file 1 [file Presentation_1.ZIP › Figure S1.pdf]

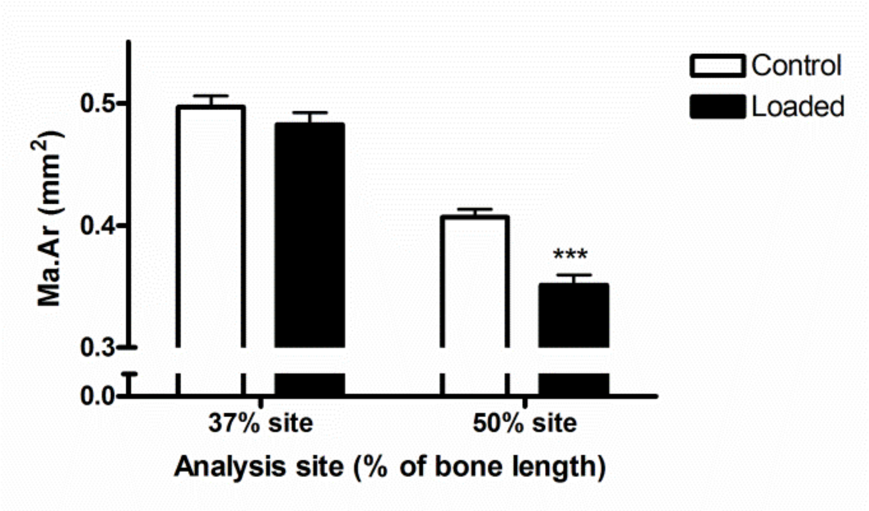

Supplementary Figure 1: Comparison of the effect of loading on Ma.Ar at the 37% and 50% site. Ma.Ar was calculated at the 37% and 50% site in the control and loaded limbs of young mice using Site Specificity software, n = 15. Bars represent the mean ± SEM. ***p<0.001 paired t-test relative to the control bones from the same mice at the same site.
