# Supplementary material for: Quantification of Alterations in Cortical Bone Geometry Using Site Specificity Software in Mouse models of Aging and the Responses to Ovariectomy and Altered Loading
Source: Front Endocrinol (Lausanne). 2015 Apr 23;6:52. doi: 10.3389/fendo.2015.00052 (PMC4407614; doi:10.3389/fendo.2015.00052)
Supplement: Supplementary file 1 [file Presentation_1.ZIP › Figure S2.pdf]

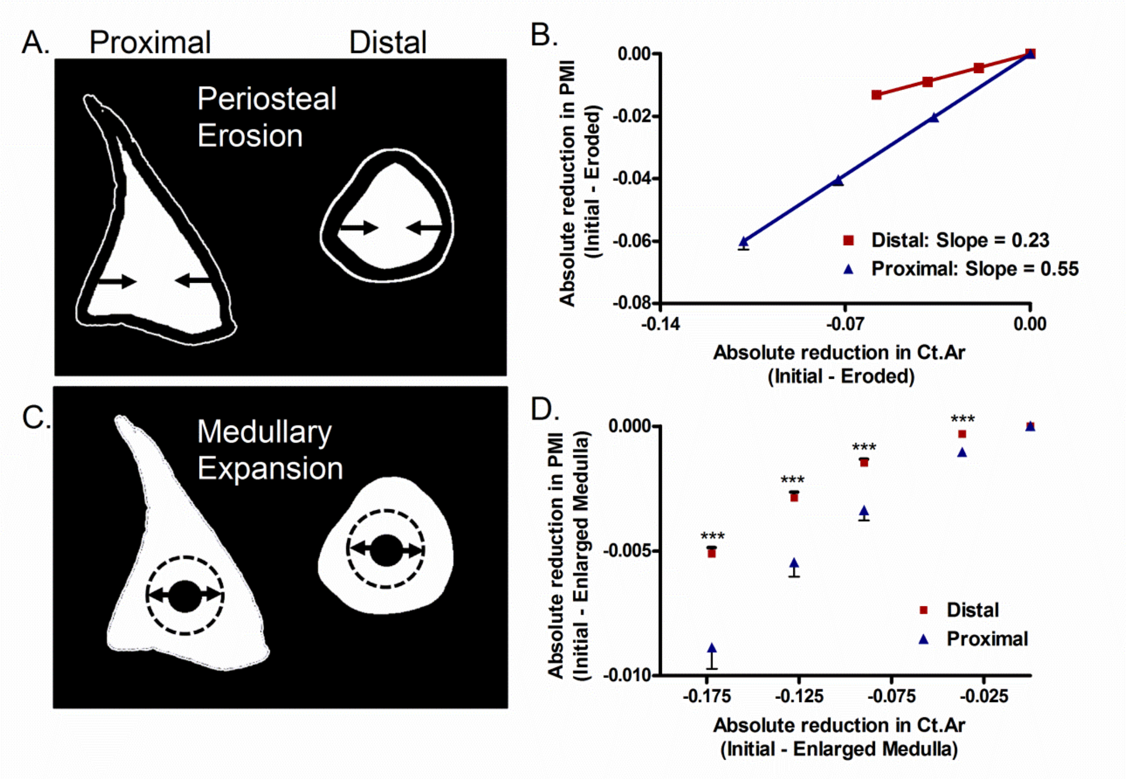

Supplementary figure 2: Simulated alterations in periosteal or endosteal dimensions alter polar moment of inertia in the proximal tibia to a greater extent than in the distal tibia. A) Reduction in periosteal dimensions were modeled by eroding the binarized image of the proximal 37% and distal 75% sites from the left tibiae of 15 mice. An exaggerated schematic is shown representing erosion of the periosteum in the direction indicated by the arrows by 10 pixels rather than the maximum of 4 pixels modeled in the graph, B, with each point representing a 1 pixel erosion all the way around the bone perimeter. B) The absolute decrease in bone area in the distal region with each modeled erosion is smaller than in the proximal region due to the smaller surface area. The smaller slope of the regression lines shown indicates that each unit of reduction in bone area results in a greater loss of PMI proximally than distally. C) Expansion of the medullary cavity was modeled by simulating expansion of a circular cavity in the direction indicated by the arrows within binarized image of the proximal 37% and distal 75% sites from the left tibiae of 15 mice. The absolute reduction in bone area was the same, not proportional to initial area. D) Equal reduction in bone area due to progressive medullary expansion results in greater absolute loss in PMI proximally than distally. Points represent the mean ± SEM. ***p<0.001 versus proximal.
